# Factors determining outcomes in adult patients operated for congenital heart diseases

**DOI:** 10.1186/1749-8090-10-S1-A325

**Published:** 2015-12-16

**Authors:** Sachin Talwar, Manikala Vinod Kumar, Shiv Kumar Choudhary, Balram Airan

**Affiliations:** 1 Department of Cardiothoracic and Vascular Surgery, Cardiothoracic Sciences Centre, India Institute of Medical Sciences, Ansari, Nagar, New Delhi, 110029, India

## Background/Introduction

Older patients with congenital heart disease (GUCH) present a unique challenge.

## Aims/Objectives

To analyze factors predicting early cardiac morbidity in GUCH at a tertiary care centre.

## Method

Between January 2004-December 2014, 1432 patients ≤13 years of age underwent surgery for GUCH. Factors associated with early cardiac morbidity were analyzed.

## Results

On multivariate analysis, previous sternotomy, aortic cross clamp time more than 45 min, cyanosis, emergency procedure were identified as independent predictors of early cardiac morbidity with respective odds ratios of 10.5, 3.7, 2.3 and 8.0. These four variables together could discriminate 77% of all procedures correctly as to their immediate post-operative morbidity. Taking the log odds with each of these 4 as the respective weights, a score was generated. The weights were previous sternotomy (2.4), aortic cross clamp > 45 min (1.3), emergency (2.1), cyanosis (0.8), if the respective condition is present, zero otherwise. The score ranged from 0 to 4.5.The average value of the score based on the 4 variables was significantly higher in cases with cardiac morbidity (0.75 ± 0.88) v/s (1.85 ± 1.17), p < 0.001. Distribution of the scores was significantly different between patients with and without morbidity. 67% patients without any morbidity had score < 1 compared to 24.6% with morbidity. Only 1.2% patients without morbidity had score of ≤ 3 compared to 15% patients with morbidity. Compared to patients having score < 1, patients with score between 1 and 2 had an odds ratio of 3.5; patients with score between 2 and 3 had an odds ratio of 6.3; > 3 had an odds ratio of 32.1 for cardiac morbidity.

## Discussion/Conclusion

Surgery for GUCH can be safely performed when adequate caution is taken in presence of four independent predictors like previous sternotomy, aortic clamp time > 45 min, cyanosis, emergency procedure.

